# Associations of Circulating MicroRNAs (miR-17, miR-21, and miR-150) and Chronic Kidney Disease in a Japanese Population

**DOI:** 10.2188/jea.JE20180233

**Published:** 2020-04-05

**Authors:** Ryosuke Fujii, Hiroya Yamada, Eiji Munetsuna, Mirai Yamazaki, Koji Ohashi, Hiroaki Ishikawa, Keisuke Maeda, Chiharu Hagiwara, Yoshitaka Ando, Shuji Hashimoto, Nobuyuki Hamajima, Koji Suzuki

**Affiliations:** 1 Department of Preventive Medical Sciences, Fujita Health University School of Health Sciences, Aichi, Japan; 2 Department of Hygiene, Fujita Health University School of Medicine, Aichi, Japan; 3 Department of Biochemistry, Fujita Health University School of Medicine, Aichi, Japan; 4 Department of Clinical Biochemistry, Fujita Health University School of Health Sciences, Aichi, Japan; 5 Department of Healthcare Administration, Nagoya University Graduate School of Medicine, Nagoya, Japan

**Keywords:** chronic kidney disease, microRNA, cross-sectional study, molecular epidemiology, epigenetics

## Abstract

**Background:**

MicroRNAs (miRNAs) play crucial roles in the development of various diseases, including chronic kidney disease (CKD). Although previous studies in clinically severe patients have investigated associations between CKD and miRNAs, with particular attention on renal fibrosis, relationships in a general population have yet to be established. The aim of this study was to examine the relationship between expression level of circulating miRNAs and CKD in a middle-aged Japanese population.

**Methods:**

A final total of 513 individuals (216 men and 297 women) who participated in the health check-up program in 2012 were included in our analysis. Quantitative real-time polymerase chain reaction was used to determine expression levels of 22 miRNAs. Estimated glomerular filtration rate (eGFR) was calculated based on serum creatinine level, sex, and age. Participants with eGFR <60 mL/min/1.73 m^2^ were defined as having CKD.

**Results:**

Three different miRNAs (miR-17, miR-21, and miR-150) showed significant correlations with eGFR after Bonferroni correction and were selected for further analyses. Expression levels of miR-17, miR-21, and miR-150 miRNAs were positively associated with eGFR after adjusting for potential confounders (*P* = 0.004, 0.002, and 0.004, respectively). Logistic regression analyses showed significantly lower odds ratios for CKD (eGFR <60 mL/min/1.73 m^2^) in the highest tertile of all three miRNAs (miR-17, miR-21, and miR-150) compared with the lowest tertile (*P* = 0.003, 0.01, and 0.02, respectively).

**Conclusions:**

We found that three circulating miRNAs were significantly associated with CKD in a general Japanese population, which suggested that these miRNAs may be biomarkers for CKD among general adults.

## INTRODUCTION

MicroRNAs (miRNAs) are small non-coding RNAs with a length of 18–25 nucleotides, and regulate gene expression by binding to the 3′-untranslated region of the target mRNA. With advances in molecular biology and technology, miRNAs have been a focus of recent medical research. Approximately 3,000 species of human miRNAs have been identified to date and regulate over 30% of all human transcripts.^1^^,^^2^ Accumulated evidence has revealed that miRNAs are associated with various pathophysiological conditions^3^ and the development of diseases, such as cancer,^4^^–^^8^ cardiovascular disease (CVD),^9^^,^^10^ diabetes,^11^ liver disease,^12^^,^^13^ kidney disease,^14^ and autoimmune disease.^15^^,^^16^ In addition, previous studies have demonstrated that miRNAs are highly stable in body fluids, such as serum^17^ and urine,^18^ because protein-bound or microvesicle-derived miRNAs in blood vessels are protected from degradation. Taken together, circulating miRNAs represent candidate biomarkers to diagnose and monitor disease.^19^^–^^23^

Chronic kidney disease (CKD) has become one of the primary health concerns in Japan, as well as in developed countries around the world. The number of individuals with an estimated glomerular filtration rate (eGFR) <60 mL/min/1.73 m^2^, the cut-off value of CKD widely used in clinical settings, has reached approximately 13 million in Japan, equal to about 13% of the Japanese adult population.^24^ The development and progress of CKD relies on the combination of pathophysiological conditions, including chronic inflammation and increased cell stress, and results in renal fibrosis in the final pathological stage of CKD. Interestingly, chronic inflammation is associated with epigenetic modifications,^25^ such as DNA methylation and RNA interference (by miRNAs) via inflammation mediators. Capturing alterations of these molecules is important for preventing the progression of CKD.

Numerous studies have investigated the associations of CKD and miRNAs in clinically severe patients and experimental animals,^26^^,^^32^^–^^35^ with particular attention to renal fibrosis. The majority of previous studies have focused on and analyzed expression levels of tissue-specific miRNAs in the kidneys. Abundant evidence on the association of miRNAs and renal fibrosis has suggested that miR-21 is strongly linked to renal pathogenesis, particularly renal fibrosis.^36^ However, whether this relationship is applicable to mildly impaired patients or the general population without renal fibrosis remains to be determined. The present study, therefore, investigated the relationships between expression levels of 22 circulating miRNAs and renal function in a middle-aged Japanese population.

## METHODS

### Study subjects

A community-based health examination has been conducted in Yakumo town, Hokkaido, in the northern part of Japan, at the end of August every year. This cross-sectional study is part of the Yakumo study, a population-based prospective study conducted in this area. Information of this health examination is provided for every household by a public relations magazine in advance. The volunteers aged 39 years or older at the health-examination and residing in Yakumo Town are eligible to participate in this health examination. Those who refuse to participate in this study or those who cannot complete the lifestyle questionnaire are excluded as research subjects. A total of 556 eligible residents participated in the health examination in August 2012. Among all those participants, 33 individuals who declined to participate in this research were excluded. Two individuals were excluded due to incomplete questionnaires. Eight women who did not undergo the rapid urine test were also excluded, thereby yielding a total sample of 513 residents (216 men and 297 women) for our analysis. Written informed consent was obtained from all participants in this study. The protocol of this study was approved by the Ethics Review Committee of Fujita Health University (Approval No. 164).

### Data collection

We collected a broad range of participants’ information, including blood laboratory data, lifestyle information, cognitive function test, and ultrasound examination, during the health examination. A self-administered questionnaire regarding lifestyle information was distributed to applicants prior to the health examination. Municipal public health nurses collected and checked for missing data with interviews at the health examination site. The following four lifestyle-related variables were defined as shown below: 1) smoking status (current, ever, or never); 2) alcohol consumption (current, ever, or never); 3) exercise habit (almost none, 1–2 h/week, 3–4 h/week, or ≥5 h/week); and 4) current medications for at least one of the four diseases of heart disease, diabetes mellitus, hypertension, or dyslipidemia (yes or no). During the health examination, urine and fasting serum samples were collected from each participant. The rapid urine test was performed to evaluate substances in urine, including protein, glucose, and erythrocytes. Collected blood samples were centrifuged within an hour of sampling and stored at −80°C until measurement. Quality-controlled biochemical analyses were performed using autoanalyzers in the laboratory of Yakumo Town Hospital. We calculated eGFR according to the equation proposed by the Japanese Society of Nephrology: eGFR = 194 × serum creatinine^−1.094^ × age^−0.287^ (×0.739 for women).^37^ According to the clinical guideline in Japan, we regarded individuals with eGFR <60 mL/min/1.73 m^2^ (CKD stage 3–5) as CKD in this study.^38^

### Measurement of microRNAs

Quantitative real-time polymerase chain reaction (qPCR) was used to detect expression levels of 22 miRNAs in sera; details of this procedure have been described elsewhere.^12^^,^^39^ Based on the previous studies, we selected these 22 miRNAs associated with metabolic phenotypes and diseases, prior to the health examination in 2012.^10^^,^^40^^,^^41^ Relative expressions of each miRNA were calculated using the comparative cycle threshold (CT) method (2^−ΔΔCT^). We used synthesize *C.elegans* miR-39 (cel-mir-39) levels as an external validation to check either the extraction of RNA or the efficacy of the cDNA synthesis.^42^^,^^43^ In this study, we used cel-miR-39 as a spike-in control in the measurement of circulating miRNAs. Although this method has several drawbacks,^44^^,^^45^ few miRNAs have been identified as an internal control in recent studies. Therefore, our method is a widely used and ideal method in the measurement of circulating miRNAs.^46^^–^^49^ We excluded 20–30 different individuals who failed in the measurement of each miRNA from our analysis. Therefore, the number of subjects included in statistical analysis differed by miRNAs.

### Statistical analysis

Normally distributed continuous variables are represented as mean and standard deviation (SD), while triglycerides (TGs) are expressed as the median (1^st^ and 3^rd^ quartile) because of the non-Gaussian distribution. The expression level of circulating miRNAs was logarithmically transformed into a normal distribution in our analyses. Pearson correlation coefficients were calculated to examine linear relationships between 22 miRNAs and eGFR. The level of significance was defined as a value of *P* < 0.05 divided by the number of comparisons based on the Bonferroni correction (2.27 × 10^−3^). Those miRNAs passing the threshold for significance were selected as plausible candidates for consecutive regression analysis. We used multiple linear regression analysis to examine the association between miRNA and eGFR. In order to estimate risk of CKD in different expression levels of miRNAs, we split participants equally into three groups “tertiles” (low, middle, and high) according to the expression level of each miRNA. Multivariable logistic regression analysis was performed to estimate odds ratios (ORs) and 95% confidence intervals (CIs) for the presence of renal dysfunction (eGFR <60 mL/min/1.73 m^2^) using the lowest tertile as a reference group. These analyses were performed after adjustment for sex, age, proteinuria, body mass index, TG, systolic blood pressure, blood glucose, smoking status, alcohol consumption status, exercise habit, and medication for non-communicable diseases. These potential confounding factors were selected based on a previous study conducted in Japan.^50^ Values of *P* < 0.05 were considered statistically significant, and all tests were two-tailed. Statistical analyses were performed using R version 3.5.0 statistical software (R Foundation for Statistical Computing, Vienna, Austria).

## RESULTS

Basic characteristics of participants stratified by CKD status are shown in Table 1. Mean ages of participants were 65.6 (SD, 9.5) years in men and 63.3 (SD, 9.4) years in women. Significant differences between normal kidney function (*n* = 395) and those who had CKD (*n* = 118) were observed only in age and medications for metabolic syndrome (both *P* < 0.001).

**Table 1.** Basic characteristics of participants (*n* = 513)

	Normal renal function (*n* = 395)	CKD (*n* = 118)
	Mean (Standard deviation)	
Age, years	63.0 (9.4)	68.5 (8.7)
Body mass index, kg/m^2^	23.5 (3.3)	23.9 (3.2)
Blood glucose, mmol/L	5.13 (0.99)	5.18 (0.97)
Systolic blood pressure, mm Hg	134.4 (19.2)	135.4 (18.7)
Diastolic blood pressure, mm Hg	76.4 (12.2)	75.8 (12.4)
Triglycerides,^a^ mmol/L	1.02 [0.73, 1.41]	1.06 [0.79, 1.43]
HDL-cholesterol, mmol/L	1.55 (0.36)	1.51 (0.35)
eGFR, mL/min/1.73 m^2^	75.6 (11.4)	50.8 (9.2)

	Frequency (%)	Frequency (%)

Smoking status		
Never	200 (50.6%)	73 (61.9%)
Ever	133 (33.7%)	33 (28.0%)
Current	62 (15.7%)	12 (10.2%)
Alcohol consumption		
Never	219 (55.4%)	80 (67.8%)
Ever	18 (4.6%)	3 (2.5%)
Current	158 (40.0%)	35 (29.7%)
Exercise habits		
Almost none	241 (61.0%)	64 (54.2%)
1–2 h/w	89 (22.5%)	23 (19.5%)
3–4 h/w	32 (8.1%)	19 (16.1%)
>5 h/w	33 (8.4%)	12 (10.2%)
Medication for non-communicable diseases^b^	169 (42.8%)	83 (70.3%)

eGFR, estimated glomerular filtration rate; HDL, high-density lipoprotein.

^a^Values are summarized as median [1st quartile, 3rd quartile].

^b^Heart disease, diabetes mellitus, hypertension, or dyslipidemia.

### Correlations between 22 miRNAs and eGFR

**Figure 1.**
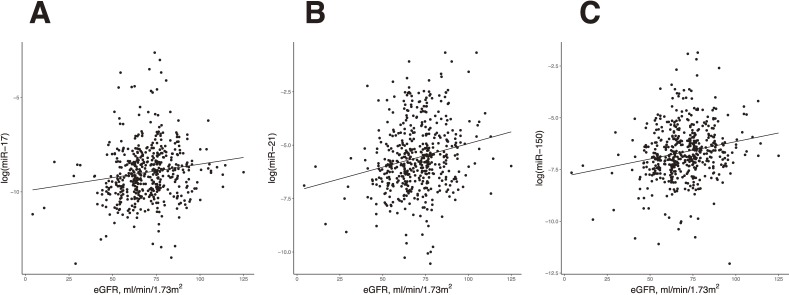
Scatter plots for the associations of target miRNAs and eGFR. *A*: miR-17, *B*: miR-21, and *C*: miR-150. eGFR, estimated glomerular filtration rate; miRNA, microRNA.

Pearson correlation coefficients between miRNAs and eGFR are summarized in Table 2. Three different miRNAs (miR-17, miR-21, and miR-150) correlated significantly with eGFR after Bonferroni correction and were selected for regression analyses. Figure 1 shows positive correlations between the log-transformed values of three miRNAs and eGFR.

**Table 2.** Pearson’s correlation coefficients for the associations between microRNAs^a^ and eGFR

microRNA	*r*	*P*-value^b^
let7d	0.091	0.05
miR-1	−0.025	0.69
miR-17	0.145	1.37 × 10^−3^
miR-20a	0.027	0.55
miR-21	0.203	7.04 × 10^−6^
miR-27a	−0.013	0.77
miR-34a	0.012	0.82
miR-92	0.060	0.18
miR-103a	−0.044	0.33
miR-122	0.122	0.01
miR-126	0.118	0.01
miR-130a	−0.013	0.78
miR-133a	−0.021	0.66
miR-146	0.109	0.02
miR-150	0.192	1.85 × 10^−5^
miR-192	0.047	0.30
miR-195	0.045	0.32
miR-197	0.08	0.08
miR-199	−0.013	0.77
miR-221	−0.033	0.46
miR-222	0.026	0.57
miR-320	0.083	0.07

eGFR, estimated glomelular filtration rate.

^a^Expression level of microRNAs was used after logarithmic transformation.

^b^*P*-value less than 2.27 × 10^−3^ was defined as statistically significant.

### Multivariable linear regression between three miRNAs and eGFR

In the multivariable linear regression analyses, selected miRNAs (miR-17, miR-21, and miR-150) were positively associated with eGFR, with respective standardized β values of 0.121, 0.134, and 0.123 (*P* = 0.004, 0.002, and 0.004, respectively), indicating that expression levels of these miRNAs were greater with normal kidney function (Table 3).

**Table 3.** Linear regression analysis for the association of circulating microRNAs^a^ with eGFR

microRNA	Standardized β	*P*-value
miR-17	0.121	0.004
miR-21	0.134	0.002
miR-150	0.123	0.004

eGFR, estimated glomerular filtration rate.

Linear regression analyses were performed after adjusting for sex, age, proteinuria, body mass index, systolic blood pressure, triglyceride, blood glucose, smoking status, alcohol consumption, exercise habit, and medication for non-communicable diseases.

^a^Expression level of microRNAs was used after logarithmic transformation.

### Odds ratio for risk of renal dysfunction

Table 4 shows adjusted ORs with 95% CIs for risk of renal dysfunction according to miRNA level. Logistic regression modeling indicates that the highest tertile in three miRNAs had significantly lower ORs of renal dysfunction compared with the lowest tertile. We also confirmed the significant linear trends of ORs for renal dysfunction according to tertiles of the three miRNAs (miR-17, miR-21, and miR-150; *P* = 0.003, 0.01, and 0.02, respectively).

**Table 4.** Logistic regression analysis for the association of circulating microRNAs^a^ with CKD (eGFR <60 mL/min/1.73 m^2^)

		miR-17 (*n* = 484)		miR-21 (*n* = 484)		miR-150 (*n* = 493)	
		OR (95% CI)	*P*-value	OR (95% CI)	*P*-value	OR (95% CI)	*P*-value
miRNA	Low	1.00	—	1.00	—	1.00	—
	Middle	0.42 (0.23, 0.73)	0.003	0.71 (0.41, 1.20)	0.20	0.71 (0.41, 1.22)	0.21
	High	0.42 (0.24, 0.75)	0.004	0.47 (0.26, 0.85)	0.01	0.49 (0.27, 0.88)	0.02

CI, confidence interval; eGFR, estimated glomerular filtration rate; OR, odds ratio.

Logistic regression analyses were performed after adjusting for sex, age, proteinuria, body mass index, systolic blood pressure, triglyceride, blood glucose, smoking status, alcohol consumption, exercise habit, and medication for non-communicable diseases.

^a^Expression level of microRNAs was used after logarithmic transformation.

## DISCUSSION

This study examined the association between circulating miRNAs and eGFR-based renal function in a middle-aged Japanese population. We found that expression levels of three different miRNAs (miR-17, miR-21, and miR-150) were significantly associated with eGFR after adjusting for potential confounders. Furthermore, the highest expression group in three miRNAs had a lower OR for renal dysfunction compared with the lowest expression group.

Previous studies have suggested that miR-21 may play a profibrotic role in the field of nephrology.^26^^–^^35^^,^^51^^–^^53^ Glowacki et al found a strong upregulation of miR-21 levels in the kidneys of mice with unilateral ureteral obstruction and patients with severe kidney fibrosis.^26^ Other previous studies observed increased miR-21 levels was associated with renal fibrosis in animal models or in patient groups.^28^^,^^33^^,^^34^ Although previous studies largely focused on tissue miR-21 expression and fibrosis and have been conducted in patients, they did not examined the association of circulating miR-21 level and renal function among healthy adults. Therefore, we tested the hypothesis that circulating miR-21 levels are associated with renal function. Contrary to our expectation, the results showed that circulating miR-21 level was positively associated with kidney function. This result was accordance with only a study regarding to the associations between miRNAs and renal function. Even though few previous studies have been reported in the field of nephrology, one possible reason for this discrepancy between our results and previous studies is that the anti-inflammatory properties of miR-21 could play a protective role in the kidney.^54^

Previous studies have shown that up-regulation of miR-21 silenced phosphatase and tensin homolog deleted on chromosome10 (*PTEN*) and programmed cell death protein 4 (*PDCD4*) as targeted genes.^55^ Inhibition of these tumor suppressor genes resulted in decreased levels of nuclear factor-kappa beta (NF-kβ), tumor necrosis factor alpha (TNF-α), and interleukin 6 (IL-6), and increased levels of IL-10, which eventually accounts for an anti-inflammatory function of miR-21.^56^^,^^57^ Considering this evidence in molecular biology, a conceivable mechanism underlying our results could be that higher miR-21 expression level induced lower inflammatory cytokines through targeting *PTEN* and *PDCD4* and consequently linked to lower inflammation and normal kidney function. Future works should focus on the relationship between expression level of circulating miR-21 and other inflammatory cytokines, which could get to the heart of this association between miR-21 and CKD in a population with normal kidney function. Another speculations for the decreased miR-21 levels in CKD was that miR-21 could be associated with increased podocyte loss.^58^ A previous animal study suggested that loss of podocytes resulted in glomerulosclerosis in mice.^59^ Taken these findings together, miR-21 may play a protective role in glomerular injury, although it is impossible to assess the causality between miR-21 and glomerular injury in our study.

Few previous studies have focused on the relationship between kidney function and other miRNAs (miR-17 and miR-150). A wide range of functions in immune activation have been identified for miR-17.^60^ A case-control study using samples from the Atherosclerosis Risk in Communities (ARIC) Study demonstrated that miR-17 was downregulated in CKD cases with hypertension compared with non-CKD cases with hypertension,^61^ indicating that lower expression of miR-17 in CKD cases induced lower immune activation. Immune dysfunction among end-stage renal disease patients is well established.^62^ The decline of kidney function seems to be continual. Immune dysfunction could, thus, play a role in the early stages of CKD. Similarly, miR-150 was highly expressed in renal biopsies from lupus nephritis patients, which might induce increased profibrotic molecules by downregulating suppression of cytokine signaling 1, an anti-inflammatory protein.^32^ However, to the best of our knowledge, the roles of miR-17 and miR-150 on CKD in general populations have yet to be systemically examined.

Three selected miRNAs examined in this study showed inconsistent associations with results from previous studies. These discrepancies may be attributable to differences in the study population (disease severity and presence of comorbidities). We have assumed that much smaller numbers of participants with renal fibrosis might have been included in our analysis compared with previous studies, because this study was conducted as a part of a health check-up in a community-dwelling population. Indeed, different functions of miR-21 were demonstrated in either normal or injured kidneys.^63^ Further work is expected to determine the relationships between circulating miRNA expression level and mildly impaired renal function in a population-based study.

A key strength of our study was that we reported significant associations between three circulating miRNAs and CKD in a general population. However, the present study also has some limitations that require discussion. First, the study design of this research was cross-sectional, making the direction of causality impossible to assess. Accordingly, further longitudinal studies with a large sample size could shed light on the causal relationships between circulating miRNAs and CKD in a general population. Second, our study participants were all Japanese, so this relationship may not be generalizable to racially different populations with diverse environmental factors, dietary habits, and lifestyles.

### Conclusions

The present study suggested that three circulating miRNAs are associated with CKD in the Japanese population. These circulating miRNAs may be novel biomarkers for CKD among general adults.
